# Resilience in Physically Abused Children: Protective Factors for Aggression

**DOI:** 10.3390/bs5020176

**Published:** 2015-04-27

**Authors:** Megan R. Holmes, Susan Yoon, Laura A. Voith, Julia M. Kobulsky, Stacey Steigerwald

**Affiliations:** 1 Jack, Joseph and Morton Mandel School of Applied Social Sciences, Case Western Reserve University, 11235 Bellflower Road, Cleveland, OH 44106-7164, USA; E-Mails: shy13@case.edu (S.Y.); jmn85@case.edu (J.M.K.); sns36@case.edu (S.S.); 2 Helen Bader School of Social Welfare, University of Wisconsin Milwaukee, 2400 E. Hartford Avenue, Milwaukee, WI 53211, USA; E-Mail: lavoith@uwm.edu

**Keywords:** resilience, child physical abuse, aggression, well-being, prosocial behavior, longitudinal

## Abstract

Aggression continues to be a serious problem among children, especially those children who have experienced adverse life events such as maltreatment. However, there are many maltreated children who show resilient functioning. This study investigated potential protective factors (*i.e*., child prosocial skills, child internalizing well-being, and caregiver well-being) that promoted positive adaptation and increased the likelihood of a child engaging in the healthy, normative range of aggressive behavior, despite experiencing physical maltreatment. Logistic regression analyses were conducted using two waves of data from the National Survey of Child and Adolescent Well-Being (NSCAW-I). Children who were physically maltreated were more likely to exhibit clinical levels of aggressive behavior at Time 1 than children who were not physically maltreated. Children’s internalizing well-being, children’s prosocial behavior, and caregivers’ well-being were associated with lower likelihood of clinical levels of aggressive behavior at Time 1. Children’s internalizing well-being and children’s prosocial behavior remained significantly associated with nonclinical aggression 18 months later. These findings highlight the role of protective factors in fostering positive and adaptive behaviors in maltreated children. Interventions focusing on preventing early aggression and reinforcing child prosocial skills, child internalizing well-being, and caregiver well-being may be promising in promoting healthy positive behavioral adjustment.

## 1. Introduction

Aggression continues to be a serious problem among children, especially those children who have experienced adverse life events such as maltreatment (*i.e*., physical abuse, sexual abuse, and neglect) [1]. Although much of researchers’ findings indicate that maltreated children are more likely to exhibit aggressive behavior [2,3,4], a growing number of researchers have examined factors that promote positive adaptation in adverse environments [5,6,7,8]. Such research focuses on resilience in children, investigating their capacity to overcome significant risks or adverse experiences and achieve positive developmental outcomes [9]. Despite strong theoretical support for the investigation of protective factors as well as risk factors put forth by the resilience theory [10], protective factors have yet to be examined as extensively or rigorously as risk factors in relation to maltreatment. Of the studies examining protective factors in maltreated children, most have focused on adolescence [11,12] and young adulthood [13,14,15]. While these developmental periods are important, investigating protective factors relevant for young children (*i.e*., 4–10 years) is critical to understanding the development of resilient functioning in children who are currently experiencing or have recently experienced physical maltreatment. Younger children may be particularly vulnerable to the effects of maltreatment [2]. Furthermore, the early development of aggressive behavior in children can lead to persistent patterns of maladaptation through adolescence and adulthood [16,17], heightening the importance of understanding early adaptation patterns. Therefore, this study included a sample of children reported to Child Protective Services (CPS) for maltreatment and investigated potential protective factors that promoted positive adaptation and increased the likelihood of a child engaging in the healthy, normative range of aggressive behavior, despite experiencing physical maltreatment. 

### 1.1. Physical Maltreatment and Aggression

Previous studies have documented a significant link between physical maltreatment and aggressive behavior in early to middle childhood [3,18,19]. Physically maltreated children are at a heightened risk for developing externalizing behavior problems, including aggression and disruptive behaviors, compared with nonmaltreated children [2,4]. Higher levels of verbal and physical aggression in physically maltreated children compared with nonmaltreated children have consistently been reported across multiple informants, including the child self-report [3], caregivers [20], and teachers/professional raters [2,4,21]. Some studies have indicated that the association between childhood physical maltreatment and aggression remains significant even after controlling for other types of maltreatment and exposure to violence [18,22]. In addition to developing aggressive externalizing behaviors, childhood maltreatment or neglect has been found to be associated with later delinquency in life [23]. Studies suggest that delinquent behavior occurs 47% more often among maltreated children than among their nonmaltreated peers [24]. 

### 1.2. Resilience to Physical Maltreatment

Although children who experience maltreatment have an increased risk for developing emotional, behavioral, and social adjustment problems [25], some maltreated children do not exhibit these negative outcomes. Results of longitudinal studies of resilience in maltreated children indicate that approximately 10%–20% of maltreated children exhibit resilient functioning [5,26,27]. Resilience is defined as the capacity for successful adaptation in the face of adversity [28]. Developing over the last several decades, resilience theory focuses on the protective processes that promote well-being and protect against risk [29]. Maintaining that resilience emerges from complex interactions among a myriad of systems in a person’s life, resilience theory highlights the role of developmental timing on the manifestation of resilience in children [30]. As such, resilience is a dynamic developmental process.

Protective factors promote resilience in maltreated children; that is, protective factors can outweigh the risk factors within children’s environments, allowing some children to achieve healthy and positive adaptations despite risk and adversity. Protective factors refer to characteristics that are associated with a lower likelihood of problems or negative outcomes [31]. Various protective factors at the individual and family level that may buffer the risk of maladaptive behaviors and promote successful behavioral adjustment for maltreated children have been identified in empirical studies (for a comprehensive review on protective factors associated with resilience in maltreated children, see Afifi and MacMillan, 2011) [32]. Furthermore, findings from several longitudinal studies indicate that protective factors may have enduring effects in promoting resilience in children [8,33,34,35]. 

Some potential protective factors that may promote resilience in maltreated children include prosocial skills, child internalizing well-being, and caregiver well-being. At the individual level, possessing prosocial skills (e.g., self-control, cooperation, assertion, responsibility) has been identified as a protective factor associated with resilience to externalizing behavior problems [7,33,36]. In a longitudinal study spanning from infancy to preadolescence, prosocial skills emerged as a significant protective factor against aggression [30]. Specifically, most children with better-developed prosocial skills had low or moderate physical aggression trajectories as opposed to high physical aggression trajectories [30]. 

Child internalizing well-being is another individual-level protective factor that has been associated with resilience in children who have experienced maltreatment. Internalizing well-being can be defined as the absence of internalizing behavior problems, which include a child being withdrawn or experiencing somatic problems, anxiety, or depression. Researchers have identified internalizing behavior problems as a risk factor for aggressive behavior in physically abused children and other high-risk and clinical samples [37,38,39]. Moreover, longitudinal designs have found internalizing well-being to have protective effects on aggressive behavior among maltreated children [35]. Other longitudinal research, however, has found concurrent but not predictive effects of internalizing symptoms on dating aggression perpetration/victimization in sexually abused adolescents [40] and that depression predicted trajectories of consistent aggression in African American but not Hispanic youths [41]. Furthermore, in the context of an intervention study, higher levels of co-occurring internalizing problems predicted larger decreases in externalizing behaviors, suggesting protective effects of high internalizing well-being, or the absence of internalizing problems, in some circumstances [42]. 

In addition to individual protective factors, a number of familial and relational protective factors can contribute to resilience among maltreated children. Caregiver well-being (*i.e.*, the absence of parental substance-use problems, depression, and other mental health problems) has been identified as a protective factor that buffers the negative effects of child maltreatment on later behavior problems [43] and prevents the development of aggression in high risk children [34]. For instance, the absence of maternal drug dependence [44], few symptoms of mental health problems [6], and less maternal depressive symptoms [34,45] have been linked to lower levels of externalizing problems in maltreated and other high-risk children [45]. Yet, little research has focused on protective factors within maltreated populations. Most research has examined protective factors in maltreated *versus* nonmaltreated children [7,35,36,38,43,44] or in other high-risk populations [33,34,37,39,41,42]. Scarce studies have examined resilient *versus* non-resilient maltreated children [6] or protective factors within exclusively maltreated groups [40,45].

The current study seeks to address gaps in the literature regarding the effects of individual and familial-level protective factors on aggression in relation to physical abuse (*i.e*., as opposed to other forms of maltreatment) among school-aged children who have been investigated by the child welfare system. Drawing from resilience theory that highlights the interaction of multiple systems in a child’s life and the role of developmental timing on the manifestation of resilience, this study examined the likelihood that a child would exhibit clinical levels of aggression in relation to his or her internalizing well-being, prosocial skills, caregiver’s well-being, and experience of physical maltreatment. This study first contributes to the literature by examining these potential protective factors effects on concurrent aggression and longitudinally on changes in aggression across an 18 month time period. Second, this study contributes to the literature by examining protective factors that promote resiliency to aggression in a maltreated sample. It was hypothesized that children who experienced physical maltreatment would be more likely to exhibit current clinical levels of aggressive behavior. Children who had higher levels of internalizing well-being and prosocial skills and whose caregivers had higher well-being would be less likely to exhibit current clinical levels of aggressive behavior. It was also hypothesized that the protective variables (the child’s internalizing well-being, the child’s prosocial skills, and the caregiver’s well-being) would remain a significant predictor of children being less likely to exhibit clinical levels of aggressive behavior 18 months later.

## 2. Method

### 2.1. Sample and Data

A secondary data analysis was conducted using data from the National Survey of Child and Adolescent Well-Being (NSCAW-I) [46]. The full NSCAW-I sample included 5501 children who had been investigated for maltreatment by CPS. Data were gathered from children, caregivers, teachers, and CPS caseworkers through in-person and telephone interviews. Families were interviewed at four time points, including after the close of the CPS investigation (baseline/Wave 1), and 18 months (Wave 3), 36 months (Wave 4), and 59–72 months (Wave 5) post-baseline. The current study included data from Wave 1 and Wave 3, which will be referred to as Time 1 and Time 2, respectively. 

The study sample includes 1207 children who were between the ages of 4 and 10 years old at Wave 1. Approximately half of the children were male and White/Non-Hispanic. The average age of children at Time 1 was 6.94 years (*SD* = 1.99) and 8.24 years (*SD* = 2.06) at Time 2. Over 65% of the caregivers were younger than 35 years old and nearly 70% had graduated high school or received some schooling beyond high school. At Time 1, 26% of the children exhibited clinical levels of aggressive behavior, whereas 22% of the children exhibited such behavior at Time 2. Table 1 displays the sample demographics.

**Table 1:** Characteristics of the sample (n = 1207).

	M (SD)/%	Range
Caregiver’s age		
Less than 35 years	65.95	
35 years or more	34.05	
Caregiver’s education		
Less than high school	30.15	
High school graduate	44.66	
High school plus	25.19	
Child’s gender (male)	51.04	
Child’s race/ethnicity		
White/Non-Hispanic	48.80	
Black/Non-Hispanic	25.68	
Hispanic	18.72	
Other/Multiracial	6.79	
Physical maltreatment reported	31.58	
T1 Child’s age	6.94 (1.99)	4–10
T2 Child’s age	8.24 (2.06)	5–12
Child’s well-being score	5.38 (1.29)	0–6
Child’s prosocial score	89.96 (16.10)	48–130
Caregiver well-being score	5.38 (1.45)	0–7
T1 Clinical level of aggression	26.68	
T2 Clinical level of aggression	22.04	

### 2.2. Measures

#### 2.2.1. Aggressive Behavior 

Aggressive behavior was reported by the child’s mother using the Aggressive Behavior Problem Scale of the Child Behavior Checklist (CBCL) [47] at Time 1 and 18 months later at Time 2. The scale included 20 questions for children aged 4 years and older. Scores were summed and converted to *t* scores. Scores between 65 and 69 signify clinical concern and scores of 70 or above signify clinical impairment [47]. Internal consistency for the aggressive behavior problem scale was 0.89 at Time 1 and 0.92 at Time 2. The *t* scores were recoded into a dichotomous variable for each time point, clinical aggression, with scores in the normal range = 0 indicating resilience and scores in the clinical concern or clinical impairment range = 1 indicating nonresilience. 

#### 2.2.2. Maltreatment: Physical Abuse 

Caseworkers responded *yes* (1) or *no* (0) to whether physical maltreatment was reported to CPS for investigation.

#### 2.2.3. Child’s Internalizing Well-being 

Child’s well-being was based on the internalizing problem behavior of the CBCL [47], which includes the Withdrawn, Somatic Problems, and Anxious Depressed Problem Scales. For children aged 4 and older, the Withdrawn Scale included 9 questions, the Somatic Problems Scale included 9 questions, and the Anxious Depressed Scale included 14 questions. Scores for each problem scale were summed and converted to *t* scores. The three problem scale *t* scores were then summed to create the internalizing problem behavior score. Child’s internalizing well-being was recoded to equal 1 if scores were in the normal range (below 63) and 0 if scores signified clinical concern (63 or above). Internal consistency for the internalizing behavior problem scale was 0.96.

#### 2.2.4. Child’s Prosocial Behavior 

Children’s prosocial skills were measured by the Social Skills Rating System (SSRS) [48]. Caregivers reported their perception of their child’s prosocial skills related to four domains: cooperation, assertion, responsibility, and self-control. The SSRS contained 39 items for children 3 to 5 years old and 38 items for children 6 years or older. Items were on a 3-point scale (1 = *never*, 2 = *sometimes*, 3 = *very often*). Scores were summed and standardized. Reliability coefficients for the current study were 0.90 for both age groups.

#### 2.2.5. Caregiver Well-being 

Caregiver well-being was a composite score created from three scales of the Composite International Diagnostic Interview Short Form (CIDI-SF) [49]. The three scales measured caregiver’s depression, alcohol use, and drug use. For depression, caregivers were asked three qualifying questions followed by five symptom questions based on the *Diagnostic and Statistical Manual of Mental Disorders-IV* criteria for major depressive episode [50]. Mothers were coded as either having a major depressive episode in the past year (0), feeling depressed but having no major depressive episode in the past year (1), or not feeling depressed (2). Internal consistency was low for the major depressive episode scale (α = 0.43). However, concordance with clinical diagnoses has been reported to range from 0.76 to 0.84 [51]. For alcohol use, caregivers were asked one qualifying question followed by seven symptom questions based on the *DSM-IV* criteria for alcohol dependence [50]. Caregivers were coded as alcohol dependent (0); heavy drinker if they reported drinking four or more drinks in one day but did not meet the criteria for alcohol dependence (1); light drinker if they reported three or fewer drinks in one day but did not meet the criteria for alcohol dependence (2); or abstainer if they reported no drinks in the past year (3). Internal consistency for heavy alcohol use yielded an alpha of 0.78. For drug use, caregivers were asked one qualifying question followed by seven symptom questions based on the *DSM-IV* criteria for substance dependence [50]. Caregivers were coded as substance dependent (0); used at least 1 drug in the past year but not substance dependent (1); or abstainer if they reported no drug use in the past year (2). Internal consistency for drug use yielded an alpha of 0.84. The three scales were then summed to represent caregiver well-being, where lower scores meant poorer well-being.

#### 2.2.6. Control Variables 

Mother’s and child’s ages and child’s gender and race were controlled for in this study. Child’s race/ethnicity was coded into four groups: White/Non-Hispanic, Black/Non-Hispanic, Hispanic, and Other. The Other category included American Indian, Native American, Asian, Pacific Islander, and Multiracial. 

### 2.3. Analyses

Two logistic regression models were fit to the data to test the research hypotheses. Logistic regression analysis is used to assess the effect of multiple explanatory variables, which can be categorical or continuous, on a discrete outcome. This analysis estimates the odds, or likelihood, of an outcomes occurring. Of interest is estimating the effect of risk and protective factors on clinical levels of aggression. Model 1 tested the likelihood that a child would exhibit clinical levels of aggressive behavior at Time 1. Model 2 tested the likelihood that a child would exhibit clinical levels of aggressive behavior at Time 2 while controlling for Time 1 aggressive behavior. All models estimated the factors that influence aggressive behavior. 

## 3. Results

Table 2 displays the results of the logistic regression analyses. The logistic regression model for Model 1 was statistically significant, χ^2^(9) = 382.68, *p* < 0.001. The goodness-of-fit Hosmer-Lemeshow (H-L) test yielded a χ^2^(8) of 9.00 and was not significant (*p* > 0.05), suggesting that the model fit the data well. According to Model 1, the lower the child’s age, the more likely the child would exhibit clinical aggression at Time 1. Compared with White/Non-Hispanic children, Hispanic children were less likely to exhibit clinical levels of aggressive behavior at Time 1. Children who were physically abused were 1.45 times more likely to exhibit clinical levels of aggressive behavior at Time 1 than children who were not physically abused. Child’s internalizing well-being, child’s prosocial behavior, and caregiver’s mental well-being were associated with less likelihood of exhibiting clinical levels of aggressive behavior at Time 1. Child’s gender was not significantly associated with clinical level of aggression. 

The logistic regression model for Model 2 was also statistically significant, χ^2^(10) = 272.34, *p* < 0.001. The H–L test yielded a χ^2^(8) of 15.05 and was not significant (*p* > 0.05), suggesting that the model fit the data well. According to Model 2, if a child was in the clinical range for aggressive behavior at Time 1, that child was 4.6 times more likely to remain in the clinical range of aggressive behavior at Time 2. Child’s internalizing well-being and child’s prosocial behavior were associated with a less likelihood of exhibiting clinical levels of aggressive behavior at Time 2. All other variables (*i.e.*, child’s gender, age, race, physical maltreatment, and caregiver well-being) were not significantly associated with children’s clinical level of aggression.

**Table 2:** Summary of logistic regression analysis for variables predicting children’s clinical level of aggression (n = 1207).

Predictor	Model 1 Aggression at Time 1			Model 2 Aggression at Time 2		
	*B*	*SE B*	*e^B^*	*B*	*SE B*	*e^B^*
Clinical aggression at Time 1				1.53 **	0.18	4.60
Child’s gender (male)	0.03	0.16	1.03	−0.19	0.16	0.83
Child’s age in years	−0.13 ***	0.04	0.88	0.02	0.04	1.02
Child’s race						
Black/Non-Hispanic	0.14	0.19	1.15	−0.13	0.20	0.88
Hispanic	0.59 *	0.23	0.55	−0.15	0.22	0.86
Other	−0.34	0.34	0.71	−0.47	0.36	0.63
Physical maltreatment reported	0.37 *	0.17	1.45	−0.21	0.17	0.81
Child’s internalizing well-being	−2.05 ***	0.17	0.13	−0.71 ***	0.18	0.49
Child’s prosocial behavior	−0.06 ***	0.01	0.94	−0.03 ***	0.01	0.97
Caregiver well-being	−0.18 **	0.05	0.83	−0.05	0.05	0.95
Constant	7.03			1.73		
*χ^2^*	382.68 ***			272.34 ***		
*df*	9			10		

Notes: *e^B^* = exponentiated *B*. Clinical aggression; child’s gender as male; physical maltreatment; and child’s internalizing well-being coded as 1 for *yes* and 0 for *no*. Child’s race as White/Non-Hispanic is the reference category. *p < 0.05, **p < 0.01, ***p < 0.001.

## 4. Discussion

This study examined early risk and protective factors that predict the likelihood of clinical levels of aggressive behaviors over time in children who have been referred to CPS as victims of alleged abuse or neglect. Physical abuse significantly predicted aggressive behavior at Time 1. Children who were physically abused were nearly 1.5 times more likely to exhibit clinical levels of aggressive behavior at Time 1 than children who were not physically abused. The association between physical abuse and aggressive behavior in this study is consistent with previous studies that have found elevated levels of aggression among victims of maltreatment, including physical abuse [2,3,4,18,19,20,21]. An unexpected result of this study is that physical abuse was a significant predictor of aggression at Time 1 but not at Time 2. Potentially, the effects of physical maltreatment on aggressive behavior may be pertinent to the onset of aggression, while individual factors are germane to its persistence.

In concordance with resilience theory, the association between protective factors and a lower likelihood of children’s clinical levels of aggressive behavior highlights the fundamental role of protective factors in fostering positive and adaptive behaviors in maltreated children. Thus, as was expected, child’s internalizing well-being, child’s prosocial behavior, and caregiver’s well-being decreased the likelihood of exhibiting clinical levels of aggressive behavior at Time 1. In addition, child well-being and prosocial skills decreased the likelihood of exhibiting clinical levels of aggressive behavior at Time 2. These findings underline the importance of child prosocial skills, child internalizing well-being, and caregiver well-being as protective factors in clinical levels of aggressive behavior. Because few studies have examined the range of resilient functioning in a sample focusing exclusively on maltreated children, these findings are particularly relevant for professionals working within the child welfare system. That is, they emphasize the importance of child prosocial skills and internalizing well-being as protective factors in the progression of aggressive behavior in maltreated children.

The insignificance of family-level protective factors (*i.e.*, caregiver well-being) at Time 2 seems to be in line with an ecological model of behavioral adaptation, with the most proximal individual protective factors having more stable and enduring effects on aggressive behavior and its progression than family-level environmental influences. For example, prosocial skills may extend beyond development of healthy interactions with peers and include social problem-solving skills. By definition, prosocial skills include cooperation, assertion, responsibility, and self-control. These domains may allow the child to conceptualize a myriad of solutions to social problems, such as becoming adept at reading social norms, facial expressions, intentions, empathy, and having realistic expectations of social situations. This adaptation may result in the child using healthy problem-solving skills rather than resorting to aggression. Additionally, these results may reflect an important transition in development and the relative influence of protective factors. That is, family-level factors may be more relevant for children when they are in early childhood (*i.e.*, at Time 1), due to a higher level of dependence on caregivers, compared to middle childhood (*i.e.*, Time 2); these results contribute early adaptation patterns to the broader literature of resilience in maltreated children.

Although individual protective factors may have more stable influences on aggressive behavior over time, caregiver-level protective factors, as more distal protective factors that are relatively sensitive to changing environmental conditions, may not have enduring buffering effects over time. Results from a recent meta-analysis [52] support this notion and indicate that a child’s individual characteristics are more strongly related to resilience and positive functioning in the face of maltreatment, compared with his or her interpersonal relationships and environment. According to resilience theory, these results may indicate that caregiver-level protective factors may have more leverage with certain developmental timing in a child’s life, suggesting windows of opportunity for protective factors interacting across system-levels [53].

Independent of child physical abuse and protective factors, being in the clinical range of aggression at Time 1 was also significantly associated with the increased likelihood of a child remaining in the clinical aggression group at Time 2, underscoring the importance of early behavioral patterns in shaping later behavioral adjustment. Specifically, the child was 4.6 times more likely to remain in the clinical range of aggressive behavior at Time 2 if he or she exhibited clinical levels of aggression at Time 1. Our finding is in line with the developmental psychopathology perspective, which posits that successful early adaptation increases the probability of continued successful adjustment in later life and that failure in early adaptation may lead to subsequent failures and maladaptation over time [54]. Empirical studies have also yielded similar evidence, showing that high levels of persistent aggression in early childhood often remain stable over time throughout middle childhood [55,56]. The findings suggest that early behavioral adaptation and patterns can set the course for either enhanced or disrupted behavioral functioning during later life, thus making it a potential point of intervention.

Several limitations qualify the findings of the current study. First, physical maltreatment was measured as a dichotomous variable (*i.e.*, presence *vs.* absence) and did not examine other maltreatment characteristics such as frequency, severity, and chronicity of maltreatment. Additionally, child’s physical maltreatment was measured only at Time 1 and not at Time 2, limited by how the data were collected. The reliance on caseworker report for the maltreatment measure could also lead to an incomplete picture, focusing only on the instance of maltreatment leading to investigation. In interpreting the effects of physical maltreatment, it is important to keep in mind the highly victimized nature of the study sample of children being investigated by the child welfare system. The effects found for physical abuse could be the result of other types of victimization co-occurring with physical abuse (*i.e.*, polyvictimization) [53]. Second, main study variables, including all three protective factors and children’s aggression at both time points, were reported by the caregiver, which may have produced biased results. Future studies may benefit by utilizing multiple informants such as child (self), peers, and teachers. Finally, the sample composition limits the generalizability of our study findings. The study sample consisted of children referred to the child welfare system for alleged child abuse or neglect, and thus the study results may not be generalizable to nonclinical populations. On a similar note, it is unclear whether the findings of this study would persist in subpopulations, such as children in out-of-home care.

Despite the strength of the longitudinal design in this study, it is important to note that the causal direction implied by this analysis cannot be unequivocally established. Past research has suggested the possibility of reciprocal relationships among many of the study variables of focus in this analysis (e.g., physical abuse and aggression, child internal well-being and aggression) [1,57]. Future research should continue to explore the directionality of these relationships. 

The findings from the present study add to the existing knowledge of resilience in maltreated children by highlighting the important roles played by protective factors. Specifically, this study contributes to our current understanding of resilience by demonstrating the unique effects of protective factors at different levels in buffering aggressive behavior in middle childhood. In addition, this study contributes to literature by examining resilience within a maltreated sample in relation to physical abuse and by examining the effects of protective factors on aggression and its development across an 18 month time period. Interventions focusing on preventing early aggression and reinforcing child prosocial skills, internalizing well-being, and caregiver well-being may be promising in promoting healthy and positive behavioral adjustment among physically maltreated children.
